# The Timed Up and Go dual-task test’s cognitive and motor outcomes show promising test-retest reliability in older adults with perceived memory impairment

**DOI:** 10.3389/fragi.2025.1715756

**Published:** 2026-01-08

**Authors:** Niklas Löfgren, Vilmantas Giedraitis, Kjartan Halvorsen, Erik Rosendahl, Anna Cristina Åberg

**Affiliations:** 1 School of Health and Welfare, Dalarna University, Falun, Sweden; 2 CIRCLE – Complex Intervention Research in Health and Care, Department of Women’s and Children’s Health, Uppsala University, Uppsala, Sweden; 3 Department of Public Health and Caring Sciences, Geriatrics, Uppsala University, Uppsala, Sweden; 4 Department of Community Medicine and Rehabilitation, Umeå University, Umeå, Sweden

**Keywords:** dementia, gait, mild cognitive impairment, motor-cognitive dual-task, psychometrics, subjective cognitive decline

## Abstract

**Background:**

It is of utmost importance to identify older adults at risk of cognitive impairment at the earliest possible stage. Previous research supports the potential of investigating step parameters and turn duration during Timed Up and Go (TUG) during single and dual-task (TUGdt) conditions to detect subtle impairment. The aim of this study was therefore to investigate the test-retest reliability and measurement error of novel outcomes related to TUG and two TUGdt tests, TUGdt-NA (naming animals) and TUGdt-MB (reciting months in reverse order), in older adults with perceived memory impairment.

**Methods:**

Thirty-four participants (18 women, mean age 76) were included and assessed with TUG, TUGdt-NA and TUGdt-MB on two different occasions, 5–10 days apart. Tests were video recorded for data extraction of spatiotemporal step parameters and turn duration. Reliability of motor and cognitive outcomes were analyzed with intraclass correlations (ICC^2.1^), standard errors of measurement and minimal detectable change (MDC). The proportional measurement error was presented with MDC%.

**Results:**

The results showed very good reliability (ICC^2.1^ ≥ 0.85) regarding total completion times, although the measurement error and proportional measurement error (MDC%) was higher during TUGdt conditions than TUG. The reliability of cognitive outcomes during TUGdt favored TUGdt-MB (ICC^2.1^ ≥ 0.77, MDC% ≤39.8). Step length was the step parameter with highest reliability (ICC^2.1^ ≥ 0.86) and lowest proportional measurement error (MDC% ≤21.4) across conditions, whereas turn duration showed good reliability during TUG and TUGdt-MB (ICC^2.1^ ≥ 0.74, MDC%≤38.9).

**Conclusion:**

The results support the potential of including TUG and TUGdt outcomes in cognitive risk evaluations among older adults.

**Trial Registration Number:**

Uppsala-Dalarna Dementia and Gait Project | ClinicalTrials.gov, identifier NCT05893524.

## 1 Introduction

Older age is the strongest risk factor for dementia disorders, which constitute significant and global health problems (WHO, 2025). Indeed, the detrimental effects of dementia are multifold and impacts not only the affected individuals and their families, but also strains national healthcare systems (Burks et al., 2021). As there is currently no cure for the disease, early detection of dementia risk is of utmost importance in order to initiate preventive treatment and health promotion. While mild cognitive impairment (MCI) is widely considered as a potential pre-dementia syndrome, it has also been found that individuals with subjective cognitive decline (SCD) have an elevated risk of developing MCI and dementia (Mitchell et al., 2014; Mendonca et al., 2016). SCD refers to self-perceived decline in cognitive capacity that is not associated with underlying disease and can emerge up to 15 years before cognitive decline is identified through objective measures (Molinuevo et al., 2017).

Growing evidence shows that cognitive impairment coexists with motor impairment (Leroy et al., 2023; Montero-Odasso et al., 2012; Mullin et al., 2022; Verghese et al., 2013) and deviant gait has even been found to precede cognitive decline by several years (Skillback et al., 2022). Although previous research has primarily investigated potential links between motor and cognitive impairment in older adults with dementia or MCI (Zhong et al., 2025), emerging findings have also identified impaired motor performance among older adults with SCD in comparison with cognitively healthy counterparts (Knapstad et al., 2019; Åhman et al., 2021). Hence, the dual task paradigm, which entails the concurrent performance of two tasks with distinct objectives (McIsaac et al., 2015) and challenges executive functions (Yogev-Seligmann et al., 2008), may therefore be relevant in the assessment of SCD. In addition, to further investigate the added challenges of performing dual tasking in relation to either task alone (i.e., single task), dual task cost (or dual task interference) can be approximated by dividing the difference between single- and dual task performance with single task performance. Both decreased dual task performance and increased dual task cost have also been previously shown to differentiate between older adults without cognitive impairment and with mild cognitive impairment (Ramirez and et, 2021; Yang et al., 2020), whereas no differences in dual task gait outcomes were found between older adults with SCD and controls in a recent review and meta-analysis (Salzman et al., 2025). However, the majority of included studies were conducted during straight overground walking, while only one study (Åhman et al., 2021) investigated motor-cognitive dual-tasking with the more complex Timed Up and Go (TUG) mobility task (Podsiadlo, 1991) and wherein the results discriminated between individuals with and without SCD. This result may indicate that more complex motor tasks may be needed to detect subtle changes in cognitive ability, even under dual-task conditions. Indeed, TUG performance entails a combination of movement segments such as rising from a seated position, walking 3 m, performing a 180-degree turn, walking back, and sitting down again. This combination of movement sequences requires movement adaptations and challenges executive functions, which have been shown to be reduced among older adults and even more among those with cognitive impairment (Pott et al., 2022; Herman and Hausdorff, 2011).

Within an ongoing longitudinal Dementia and Gait project, two TUG dual-task (TUGdt) conditions have been developed and evaluated with regard to its potential of identifying older adults at risk of cognitive decline and dementia (Cedervall et al., 2020; Åberg et al., 2023). The test procedure was designed in close collaboration with memory clinic specialists to enable its implementation into clinical practice, and the assessment methodology has been acknowledged in a systematic review (Ramirez and et, 2021). Previous findings have been encouraging, particularly with regards to the novel TUGdt outcome *words/10 s* which not only has been associated with neurodegeneration (Åhman et al., 2019), but also been found to discriminate between controls, older adults with SCD, MCI, and dementia (Löfgren et al., 2025), as well as to predict conversion to dementia (Åberg et al., 2023). In addition, the use of an innovative method involving marker-free video recordings during the TUG test conditions enabled extraction of specific step parameters (Åberg et al., 2021), which may contribute to the investigation of subtle outcomes such as step length and turn duration. Accordingly, increasing research interest has been directed toward investigating TUG performance in a more detailed manner, including specific step performance and turn duration (Bottinger et al., 2024; Ortega-Bastidas et al., 2023). Initial results indicate from our group indicate that shorter step length (Löfgren et al., 2025) and longer turn duration (unpublished results) during TUG with a cognitive dual-task significantly discriminates between individuals with SCD and controls, whereas others have found indications that the 180-degree turn entail an added capacity for identifying a history of falls (Brauner et al., 2022). However, despite this potential importance of investigating specific step parameters and segments of TUG, few studies have investigated the reliability of these outcomes. In addition, although the reliability of the full TUG has been investigated in various populations, research regarding the reliability of the cognitive task as well as on populations with subtle cognitive impairment is lacking.

Individuals with perceived memory impairment—a population similar to individuals with SCD—are likely to undergo cognitive assessment. Hence, it is vital to investigate the reliability and measurement error of TUG and TUGdt outcomes in this population. The aim of this study was therefore to investigate the test-retest reliability and measurement error of the performance of the TUG conditions with regard to total completion time, performance of the cognitive tasks, the specific step parameters, and the duration of the 180-degree turn in older adults with perceived memory impairment.

## 2 Materials and methods

This was an observational cohort study with a test-retest design, that is part of the longitudinal UDDGait project (Trial registration number: NCT05893524). The Regional Ethical Review Board in Uppsala, and the Swedish Ethical Review Authority approved this study, and informed consent was obtained from all participants prior to study commencement.

### 2.1 Participants

Older adults with perceived memory impairment were recruited. Initial recruitment was halted midway due to the COVID-19 pandemic, therefore participants were recruited and assessed at two geographical cohorts in central Sweden. During the period 2019–2020, 16 individuals were recruited via a specialist memory clinic in connection with a healthcare visit and, in 2024, 18 individuals were recruited through collaboration with a housing facility for older people. Inclusion criteria were: ≥50 years, perceived (self-reported) memory impairment, and ability to rise from a chair and walk 3 m back and forth without the use of walking aids. Two physiotherapists (one for each cohort) with vast experience in instructing and assessing TUG in various populations acted as test administrators and followed a standard protocol regarding how to instruct and administer the tests.

### 2.2 Data collection

All participants were assessed under similar conditions during two test occasions, with an interval of 5–10 days between the tests, since it has been found that a time interval ranging from 2 days to 2 weeks is adequate for the test-retest assessment of health status instruments (Marx et al., 2003). Data collection was carried out in line with the study protocol (Cedervall et al., 2020). At the first visit, and prior to TUG assessments, a brief interview (following a standardized questionnaire) regarding perceived cognitive and physical status was conducted and demographic data were collected, including memory assessment, screening for depressive symptoms (Almeida and Almeida, 1999), and general physical ability. For the TUG data collection, the participants were instructed to rise from sitting on a standard chair with armrests, walk 3 m, turn 180°, walk back to the chair and sit down again (Podsiadlo, 1991; Almeida and Almeida, 1999). The instructions were given verbally, and the test administrator also demonstrated the test to the participants. Three conditions, recorded with two synchronized high-definition video cameras, were assessed in the following order: (WHO, 2025) TUG as a single task test (Burks et al., 2021), TUGdt-NA (TUG while simultaneously naming animals), and (Mitchell et al., 2014) TUGdt-MB (TUG while reciting months in reverse order, starting with December). Prior to each TUG-condition, participants were instructed to complete the task at comfortable speed and time was registered with a stopwatch. The timing started when the participants back left the backrest and stopped when their backside touched the chair again. During the TUGdt conditions, the test administrator recorded the correct number of animals/months recited by the participants, which were controlled for when reviewing the videos.

At the beginning of the retest occasion, the participants were again interviewed regarding their cognitive and physical status and other possible adverse events that may have occurred, between the test occasions. Following this, they underwent the TUG assessments under conditions identical to the prior test occasion.

### 2.3 Data preparation

For TUGdt-NA and TUGdt-MB, the number of correctly recited animals/months per 10 s during the test performance was calculated (recited animals or months/time*10) and documented as TUGdt-NA or TUGdt-MB words/10s. Registration of correct words recited during TUGdt-NA and TUGdt-MB was performed by reviewing the video recordings and followed the procedures used in establishing norms for such tests. Dual-task cost (DTC) was calculated as: TUGdt time–TUG time/TUG time * 100 (McIsaac et al., 2015).

Data processing for the step parameters was based on the documentation from two synchronized high-definition video cameras using a semi-automatic method aided by a technique for human 2D pose estimation of events of contact points of the heel with the ground (i.e., heel strike), based on a deep learning procedure, described in more detail elsewhere (Åberg et al., 2021), see Supplementary Material. Based on determined heel strike events, steps were quantified during gait, in two segments of straight walking: 1) during gait toward the 3-m line, starting with the second heel strike and ending with the last heel strike for which no part of the foot had passed the line, and 2) during gait back to the chair, starting with the first heel strike for which the whole foot had passed the 3-m line and ending with the last heel strike that did not appear to be part of preparations for sitting down, as indicated by a foot twist or an atypical short step. For each step parameter, the mean of all analyzed steps for each participant was used in the analysis. The time to turn (between 1 and 2) was measured from the last heel strike of gait before the 3-m line and ending at the first heel strike of gait back to the chair, as described above.

### 2.4 Analysis

Statistical analyses were conducted with SPSS (version 29, IBM Inc., Armonk, NY). Descriptive characteristics were presented with mean and standard deviations. Reliability for each outcome was investigated with intraclass correlations (ICC_agreement_, 2-way random effects = ICC 2.1) with 95% confidence. To categorize the level of ICC_agreement_, Bland-Altman’s classification was used: ˂0.20 = poor; 0.21–0.40 = fair; 0.41–0.60 = moderate; 0.61–0.80 = good; 0.81–1.00 = very good (Altman, 1991). Measurement errors were calculated in two steps. First, SEM_agreement_ was calculated as follows: SEM = √ within subject error variance (Bland and Altman, 1996). Second, to investigate the measurement error on individual level, the minimal detectable change (MDC) with 95% confidence interval (CI^95^) was calculated with the formula: 1.96 x √2 x SEM_agreement_ (Beckerman et al., 2001). To illustrate the magnitude of the measurement error, the proportional measurement error (MDC%) was calculated by dividing the MDC value with the mean result of both tests (Flansbjer et al., 2005), whereby MDC% ≤30 has been proposed to be acceptable (Para et al., 2022). Additionally, to analyze systematic differences between the test occasions Bland-Altman plots were conducted.

## 3 Results

Thirty-five older adults with perceived cognitive impairment, deriving from two geographical cohorts due to the COVID-19 pandemic, were enrolled in this study. One participant reported sickness between test sessions 1 and 2 and was therefore excluded from analyses, however all remaining participants reported stable conditions between the sessions, resulting in 34 participants included in the analyses for completion times of the TUG and TUGdt conditions. However, due to technical problems with video recordings the extraction of step parameters was not possible for three participants during TUG and TUGdt-NA, and for four participants during TUGdt-MB. In addition, one participant made several long stops during all TUG conditions, whereby steady state gait was not achieved, and was therefore excluded from the analyses of specific step parameters and turn duration.

The participants were 52–91 years old (CI^95^: 73.1–79.3), 18 were women, 17 (50%) had participated in higher education, and 18 (52%) lived with someone (see Table 1 for participant characteristics).

**TABLE 1:** Participant demographics.

Males/Females, n (%)	16 (47)/18 (53)	​
Co-residing, Y/N	18/16	​
University education, Y/N	17/17	​
​	Mean (SD)	Range
Age	76.2 (8.9)	52–91
Body height, m	1.69 (9.3)	1.53–1.87
MMSE-SR, 0–30	27.1 (2.6)	20–30
Gait speed, m/s	0.98 (2.23)	0.58–1.46
Handgrip, kg	25.4 (11.2)	4.0–63.0
GDS-4, 0–4	0.6 (1.0)	1–4

SD, standard deviation; m,meter; MMSE-SR, Swedish version of the Mini-mental State Exam; m/s, meters per second; kg, kilograms; GDS-4, 4-item version of the Geriatric Depression Scale.

The participants in the initial cohort (recruited 2019–2020) were younger (mean age 73 vs. 79 years), had a lower proportion of women (37.5% vs. 67%), higher proportion of participants living together with someone (69% vs. 39%), and smaller proportion of participants with higher education (44% vs. 56%). However, the participants from both cohorts showed similar results regarding the MMSE-SR and physical function (10-m walk speed).

### 3.1 Test-retest results

For test-retest results regarding the performance of the different TUG conditions (TUG, TUGdt-NA, TUGdt-MB, and dual-task cost for TUGdt-NA and TUGdt-MB, respectively), reliability results were categorized as very good (ICC 0.85–0.90). However, results regarding the measurement error were proportionally higher during the TUGdt-NA (MDC% = 35.5) and TUGdt-MB (MDC% = 39.1) than during TUG (MDC% = 23.0), and significantly higher for DTC (125.8%–133.7%), as presented in Table 2.

**TABLE 2:** Test-retest reliability and measurement error of the different TUG conditions (N = 34).

Parameter	Test 1			Test 2			Cronbach´s alpha	ICC^2.1^	CI^95^	SEM	MDC	MDC%
	Mean	CI^95^	Range	Mean	CI^95^	Range						
TUG (s)	13.0	11.9–14.2	6.9–21.9	12.5	11.3–13.7	7.6–26.0	0.95	0.90	0.80–0.95	1.06	2.93	**23.0**
TUGdt-NA (s)	16.7	14.2–19.1	6.8–43.9	16.0	13.7–18.2	8.1–34.7	0.95	0.90	0.82–0.95	2.09	5.79	35.5
TUGdt-MB (s)	16.8	14.3–19.3	7.5–43.9	16.2	14.0–18.3	8.3–34.0	0.94	0.88	0.77–0.94	2.33	6.45	39.1
DTC_TUGdt-NA	25.8	15.1–36.4	−8.5–157.1	27.2	15.1–39.2	−11.5–179.1	0.92	0.85	0.71–0.92	12.77	35.39	133.7
DTC_TUGdt-MB	27.2	15.9–38.5	−17.4–156.9	29.5	17.0–41.9	−5.8–172.5	0.92	0.86	0.73–0.93	12.87	35.66	125.8
Correctly recited animals	8.0	7.0–9.0	1–14	8.7	7.8–9.6	2–13	0.78	0.63	0.38–0.80	1.69	4.68	56.1
Correctly recited animals/10s	5.5	4.6–6.4	0.6–10.3	6.0	5.2–6.7	1.2–9.7	0.80	0.66	0.42–0.81	1.39	3.84	66.9
Correctly recited months	9.1	8.0–10.1	1–13	9.4	8.5–10.3	3–13	0.87	0.77	0.59–0.88	1.32	3.67	39.8
Correctly recited months/10s	6.1	5.3–7.0	0.3–10.1	6.4	5.6–7.2	1.7–10.1	0.94	0.89	0.79–0.94	0.79	2.18	34.9

TUG, timed up and go; ICC, intra class correlation, CI^95^, 95% confidence interval; SEM, standard error of measurement; MDC, minimal detectable change, MDC%, MDC/mean*100; s, seconds; TUGdt-NA, timed up and go while naming animals; TUGdt-MB, timed up and go while reciting the months of the year in reverse order, DTC, dual-task cost.

The results for the performance of the cognitive tasks were varied. While the reliability of both the number of correctly named animals and recited months (in reverse order) were categorized as good, the results were higher for correctly recited months (ICC = 0.77) than for naming animals (ICC = 0.63). An even more distinct pattern was shown for the outcomes animals/10 s and months/10 s, where the reliability of the latter was categorized as very good (ICC = 0.89) and the former as good (ICC = 0.66). The results of measurement error were similar, with markedly higher proportional measurement errors for both the number of correctly named animals (MDC% = 56.1) and animals/10 s (MDC% = 66.9) than for correctly recited months (MDC% = 39.8) and recited months/10 s (MDC% = 34.9).

Regarding step parameters during TUG, the reliability was very good for step duration, SS duration, and step length (ICC_agreement_ = 0.81–0.89), with proportional measurement errors ranging from 10.8% to 15.5% (Table 3). For DS duration and turn duration, the reliability was good (ICC_agreement_ = 0.77 and 0.74, respectively), with similar proportional measurement errors (MDC% = 41.7 and 38.9). *Step width* was found to be of moderate reliability (ICC_agreement_ = 0.56), with a proportional measurement error of 38.9%.

**TABLE 3:** Test-retest reliability and measurement error of specific step parameters and turn duration during the TUG conditions.

Parameter	N	Mean T1	CI^95^	Range	Mean T2	CI^95^	Range	Cronbach´s alpha	ICC^2.1^	CI^95^	SEM	MDC	MDC%
TUG													
Step duration (ms)	30	632.4	604.4–660.5	520.0–805.7	609.8	584.3–635.4	528.0–794.3	0.94	0.85	0.58–0.94	28.45	78.85	**12.7**
DS duration (ms)	30	148.0	131.1–165.0	66.7–268.6	130.9	116.0–145.8	64.0–230.0	0.90	0.77	0.42–0.90	21.30	59.04	41.7
SS duration (ms)	30	484.4	468.6–500.2	413.3–594.3	478.0	462.7–495.2	410.0–640.0	0.90	0.81	0.65–0.91	18.74	51.96	**10.8**
Step length (cm)	30	55.87	52.21–59.53	39.51–81.47	56 0.53	53.03–60.04	42.09–80.30	0.94	0.89	0.79–0.95	3.13	8.68	**15.5**
Step width (cm)	30	18.91	17.45–20.37	13.33–26.33	18.22	16.74–19.69	11.48–26.20	0.72	0.56	0.26–0.76	2.60	7.22	38.9
Turn duration (s)	30	3.78	3.48–4.08	1.96–5.56	3.66	3.39–3.93	2.32–5.20	0.85	0.74	0.53–0.87	0.39	1.08	**29.0**
TUGdt-NA													
Step duration (ms)	30	732.0	674.3–789.7	520.0–1274.3	717.1	671.1–763.1	540.0–1040.0	0.69	0.54	0.22–0.75	94.7	262.4	36.2
DS duration (ms)	30	199.2	168.8–229.6	66.7–417.1	181.8	157.7–205.8	64.0–330.0	0.77	0.62	0.35–0.80	45.6	126.5	66.4
SS duration (ms)	30	532.8	499.5–566.2	440.0–857.1	535.3	507.7–562.9	424.0–710.0	0.61	0.45	0.11–0.70	60.28	167.1	31.3
Step length (cm)	30	51.17	47.40–54.94	27.07–78.63	51.42	47.10–55.74	22.27–79.90	0.93	0.87	0.75–0.94	3.90	10.81	**21.1**
Step width (cm)	29	18.46	16.53–20.39	11.53–28.70	19.78	18.07–21.49	12.33–29.80	0.78	0.63	0.35–0.81	2.96	8.21	43.0
Turn duration (s)	30	4.42	3.84–4.99	2.24–11.36	4.12	3.73–4.52	2.48–6.36	0.71	0.54	0.24–0.75	0.90	2.50	58.6
TUGdt-MB													
Step duration (ms)	29	776.3	688.5–864.2	533.3–1685.0	728.5	672.5–784.6	520.0–1220.0	0.81	0.67	0.41–0.83	112.49	311.0	41.4
DS duration (ms)	29	217.0	176.1–257.9	80.0–540.0	186.0	155.5–216.5	70.0–400.0	0.86	0.72	0.46–0.86	51.40	142.46	70.7
SS duration (ms)	29	559.3	506.4–612.3	424.6–1145.0	542.5	509.8–575.3	400.0–820.0	0.76	0.62	0.33–0.80	71.64	198.59	36.1
Step length (cm)	29	50.69	46.73–54.65	22.50–76.07	50.76	46.79–54.73	23.71–69.64	0.92	0.86	0.72–0.93	3.92	10.88	**21.4**
Step width (cm)	29	18.74	16.88–20.60	12.10–28.38	18.02	16.54–19.50	11.13–25.40	0.84	0.72	0.48–0.86	2.36	6.53	35.5
Turn duration (s)	29	4.32	3.89–4.75	2.08–6.88	4.09	3.64–4.54	2.20–7.80	0.86	0.75	0.54–0.88	0.58	1.60	38.1

TUG, Timed Up and Go, ICC , intra class correlation, CI^95^, 95% confidence interval; SEM, standard error of measurement; MDC, minimal detectable change; MDC%, MDC/mean*100; ms, milliseconds; cm, centimeters; TUGdt-NA, Timed Up and Go while naming animals, TUGdt-MB, Timed Up and Go while reciting the months of the year in reverse order.

For step parameters during TUGdt-NA, *step length* was found to be of very good reliability (ICC_agreement_ = 0.87), with a proportional measurement error of 21.1%. The step parameters *DS duration* and *step width* showed similar (good) reliability (ICC_agreement_ = 0.62 and 0.63) with proportional measurement errors of 66.4% and 43.0%, respectively. Finally, the parameters step duration, SS duration, and turn duration were all of moderate reliability (ICC_agreement_ ≥ 0.45–0.54), with proportional measurement errors ranging between 31.3% and 58.6%.

The investigation of step parameters during TUGdt-MB found the reliability of *step length* to be very good (ICC_agreement_ = 0.86), with a proportional measurement error of 21.4%. For all other parameters, the reliability was found to be good (ICC_agreement_ = 0.62–0.75), with proportional measurement errors ranging from 35.5% to 70.7%.

**FIGURE 1:**
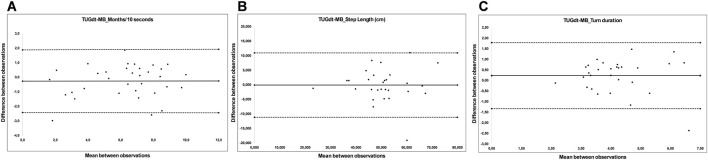
**(A–C)** Bland-Altman plots, illustrating the test-reliability of **(A)** the number of correctly recited months per 10 s, **(B)** step length (centimeters) during TUGdt-MB, and **(C)** turn duration (seconds) during TUGdt-MB. The difference between the observations is plotted against the mean of the observations. The solid line represents the difference between the two observations and the dotted lines represent the upper and lower limits of agreement.

Bland-Altman Plots (Figures 1A–C) indicate tendencies of systematic differences, where the participants performance during the retest assessments generally were slightly improved in comparison to the prior assessment. However, we did not identify any clear tendencies of heteroscedasticity.

## 4 Discussion

This is the first study to investigate the test-retest reliability of TUG and TUGdt conditions, including the cognitive task and specific step parameters, in older adults with perceived cognitive impairment. The results show very good reliability regarding completion times during the different TUG conditions, although both the measurement error and proportional measurement error were higher during the TUGdt conditions, particularly for DTC. The reliability of the cognitive outcomes during TUGdt showed that the highest reliability (good to very good) and lowest measurement error occurred during outcomes related to TUGdt-MB (correctly recited months and correctly recited months/10 s). There were discrete tendencies of systematic differences as the performance at the retest occasions was slightly improved (Figure 1), however, given the small magnitude of these differences, they were not considered to indicate learning bias.

The assessment procedures were designed to be adaptable to clinical practice and the robustness of the reliability findings for completion times of the different TUG conditions are supported by their alignment with previous results in various populations, using different assessment procedures (Rodrigues et al., 2023; Hofheinz and Schusterschitz, 2010; Smith et al., 2016). To minimize time consumption, an important factor for potential implementation (Wang et al., 2023), our procedure included a single trial for each condition. This procedure differs from other studies exhibiting variations between including/excluding practice trials, using multiple/single trials, and where data collection has been conducted in motion laboratory settings or in the participants’ homes. In addition, although the measurement errors regarding total completion times were higher than in similar studies on older adults (Donoghue et al., 2019), they were lower compared with studies on individuals with cognitive impairment (Braun et al., 2019), which may be considered reasonable due to the expected variability among individuals also with lower levels of cognitive impairment (Kwak et al., 2023). Further, while the proportional measurement errors during the investigated TUGdt conditions in our study were slightly higher than recommended levels, there is generally a lack of studies presenting this outcome, making direct comparisons challenging. The results of dual-task cost, for both the TUGdt-NA and TUGdt-MB, showed markedly higher reliability (very good) compared with what has been presented in a recent review on the reliability of dual-task cost metrics (Pike et al., 2023), as well as in similar studies investigating TUG dual-task costs in populations with intact and impaired cognition (Åhman et al., 2021; Venema et al., 2019). On the other hand, the measurement error was similar to the findings in these studies. Considering also that the proportional measurement error was well beyond recommended levels (≤30%), measures need to be taken in to control for variability to optimize measurement robustness for this outcome during TUG. However, few studies have presented MDC% for TUG with and without a dual-task, (making direct comparisons challenging). Nevertheless, one previous study (Venema et al., 2019) that included 50 individuals found higher MDC results for dual-task cost than we found, both among cognitively impaired individuals (MDC = 129) and in cognitively intact older adults (MDC = 41). In addition, a previous review that investigated the reliability of gait parameters derived from instrumented walkways (Para et al., 2022) found the MDC% for gait speed to exceed 30% (MDC% = 42) in individuals with cognitive impairment. Considering this, it may be considered somewhat unsurprising that the results on MDC% were generally higher than optimal in this study (despite the absolute MDCs being at similar levels of corresponding studies). However, more fragile groups may be expected to entail higher degrees of variability which reasonably affects MDC%. Therefore, as MDC% is an important outcome for evaluating the magnitude of the measurement error, future studies need to present MDC% to a higher extent. Not least to enable comparisons of assessment instruments in populations where larger degree of variability can be expected.

The two dual-task conditions used in the UDDGait project can be categorized as mental tracking tasks according to a proposed taxonomy of cognitive tasks during motor-cognitive dual-task investigations (Wollesen et al., 2019). Despite this, the results varied with higher reliability and lower measurement error for the TUGdt-MB outcomes in relation to TUGdt-NA. In addition, few studies have investigated the reliability, not least the measurement error, of the cognitive task during dual-task assessments, and particularly during TUG. However, results presented in a previous systematic review (including straight overground walking and TUG) indicate large variations of reliability between different tasks (Yang et al., 2015), where the tasks with the highest reliability showed similar results to what we found for TUGdt-MB. Bearing in mind that more complex tasks such as TUG may inflict larger cognitive load when conducted as a dual-task, the finding of very good reliability (combined with a proportional measurement error close to 30%) for the TUGdt-MB outcome months/10 s may be of particular importance. Indeed, this novel outcome has been found to predict conversion to dementia over a period of up to 6 years among individuals with SCD or MCI and its potential has previously received particular attention (Ramirez and et, 2021). Therefore, the indication of the robustness of the outcome months/10 s may be argued to support its potential for implementation into clinical practice.

The findings of our study regarding the reliability of step parameters and turn duration during TUG and TUGdt-MB performance showed good to very good reliability, with even better reliability for TUG as a single task. In relation to one of the few comparable studies (Smith et al., 2016), our results showed similar or higher reliability for most outcomes (whereas TUGdt-NA showed slightly worse results). In addition, especially our findings of very good reliability and acceptable proportional measurement errors for *step length* during all conditions are aligning with the findings in a recent review (Para et al., 2022) on the reliability of step parameters assessed via instrumented walkways. This finding may be of particular importance since we have previously found step length to discriminate between groups of people with different cognitive ability (Löfgren et al., 2025). Another important finding refers to *turn duration*, a parameter attributed with the potential of identifying fall risk (Brauner et al., 2022). Our results showed similar reliability (good) and measurement errors during TUG and TUGdt-MB, whereas the reliability was moderate for TUGdt-NA. Other studies investigating the reliability of the duration of 180-degree turn during TUG in older adults have generally found mixed results. However, the definition of the start and end of the turn differs between the studies (McGrath et al., 2011; Salarian et al., 2010). Whereas others have registered the turn with gyroscopes and based turn duration on angular velocity, our method was based on perceived clinical applicability. Nevertheless, to enable credible replications of results, future research needs to establish a consensus on how to register turn duration during TUG.

This was not without limitations. First of all, although the sample size was similar to related studies investigating test-retest reliability in fragile populations (Salarian et al., 2010; van Lummel et al., 2016), it was lower than what has been recommended for studies for reliability investigations (Ter et al., 2007). Therefore, the results need to be interpreted with caution and particularly the results of MDC% highlight the importance that future studies aspire to recruit larger samples when investigating subtler outcomes during challenging conditions. In addition, the assessments entailed performing TUG and TUGdt conditions once each, without a practice session, as recommended by Podsiadlo (1991), which may have caused larger measurement errors than if more than one session for each condition had taken place. For example, it has previously been suggested that nine strides are required for the assessment of dual-task gait (Hollman et al., 2011), which is rarely achievable during TUG. However, in the current UDDGait project, the assessment procedure was developed in close collaboration with expert clinicians in order to optimize potential for implementation, whereby time efficiency and minimized patient burden are crucial factors (Wang et al., 2023). Therefore, certain trade-offs may be necessary. Considering that previous research findings have shown that, particularly during TUGdt-MB, the outcome of correct words/10 s can predict conversion to dementia (Åberg et al., 2023), whereas step length can discriminate between people with different levels of cognitive function (Löfgren et al., 2025), the test-retest reliability of these outcomes support the potential of implementing this assessment into clinical practice. Nevertheless, more research is needed on the psychometric properties of specific step parameters and segments of TUG under single- and dual-task conditions, particularly in populations with perceived symptoms of cognitive impairment.

## 5 Conclusion

The results of this study show good to very good reliability for completion times of TUG, TUGdt, and the cognitive tasks, with acceptable measurement errors, particularly during TUG. For novel and potentially important outcomes such as words/10 s, step parameters, and turn duration, the reliability was generally good to very good during TUG and good during TUGdt-MB, whereas the magnitude of measurement errors varied. Particularly step length showed very good reliability with acceptable measurement during all TUG conditions, and turn duration showed good reliability during TUG and TUGdt-MB. Although more research is needed, these results support the robustness of outcomes that have previously been found to predict dementia and discriminate between different levels of cognitive function among older adults and may therefore support their potential for implementation into clinical practice.

## Data Availability

The material analyzed during the current study is not publicly available due to its content of sensitive personal data. Datasets generated can be available from the principal investigator Anna Cristina Åberg upon reasonable request, after ethical considerations. Requests to access the datasets should be directed to anna.cristina.aberg@uu.se.
